# Genome Sequences of Parvovirus B19 Reference Strains

**DOI:** 10.1128/genomeA.00830-14

**Published:** 2014-10-09

**Authors:** Jan-Hendrik Trösemeier, Annika Branting, Vladimir V. Lukashov, Johannes Blümel, Sally A. Baylis

**Affiliations:** a Paul-Ehrlich-Institut, Langen, Germany; b Thermo Fisher Scientific, Carlsbad, California, USA; c Center for Infection and Immunity, Amsterdam, the Netherlands

## Abstract

We report here the sequences of two reference strains of parvovirus B19 (B19V) used for quantitation of B19V DNA. One reference strain has been established by the World Health Organization (WHO) and the other by the European Pharmacopeia (Ph. Eur.) and belong to B19V genotype 1a1 and 1a2, respectively.

## GENOME ANNOUNCEMENT

Parvovirus B19 (B19V) is a single-stranded DNA virus belonging to the genus *Erythroparvovirus*. Diseases associated with B19V include erythema infectiosum, aplastic crisis, hydrops fetalis, and arthritis. Even in asymptomatic individuals, viremia may exceed 12 log_10_ IU/ml of B19V DNA, and consequently plasma used in the manufacture of medicinal products is screened by nucleic acid testing (NAT) to exclude units containing high titers of B19V (1). The World Health Organization (WHO) has established an international standard (IS) and an international reference panel (IRP) comprising three different genotypes of B19V which have been used in the standardization of NAT assays for both blood/plasma safety as well as for clinical diagnostic purposes (2). The 1st and 2nd ISs correspond to the genotype 1 strain included in the 1st WHO IRP, and this represents the most predominant strain circulating worldwide. Although the ISs, together with a calibrated biological reference preparation (BRP) from the European Pharmacopeia (Ph. Eur.), have been used to define the maximum threshold for B19V DNA levels (10 IU/µl) in plasma pools (3, 4), the genomic DNA sequences of these strains have not been reported.

The B19V genome sequences, excluding the inverted terminal repeats (ITRs) of ~350 nucleotides, were amplified by PCR. Amplicons were sequenced using the Ion Torrent PGM (Life Technologies GmbH, Darmstadt, Germany). Sequencing reads were preprocessed by PCR-adapter trimming using FASTA-Tools (5) and quality filtering using PRINSEQ (6). *de novo* assembly was performed with the MIRA 4 assembler (7). On the basis of BLAST searching against the NCBI database, the sequence with the highest identity and *E* score was chosen for reference-assisted scaffolding using Mira and BWA (8). Consensus sequences, based on reference-assisted scaffolding, were constructed using samtools (9).

A comparison of the two B19V reference strains revealed that they share 98.63% nucleotide sequence identity; the A+T content of both strains is 58.1%. Homopolymer tracts of 5 consecutive Cs, 7 As, 7 Ts, and 8 Gs were resolved without difficulties. In the case of the WHO strain, 9 nucleotides upstream of the nonstructural protein (NS1) start codon, a polymorphism was identified with a T present in 87% of the reads and a C in the rest. Analysis of B19V sequences in the NCBI database demonstrated that most genotype 1 B19V strains contained a C at this position (including the BRP), although a small number contain a T, which is more usually found in genotype 2 and 3 B19V strains.

Phylogenetic analysis of the two strains, using the complete NS1-VP1/2 fragment of the genome, was performed as previously described (10) and demonstrated that the WHO strain grouped with B19V genotype 1a1 and that the BRP strain grouped with genotype 1a2.

These results indicate that the 1st and 2nd WHO IS and the Ph. Eur. reference B19V strains are highly conserved genotype 1 viruses representing two separate genogroups which are both widespread throughout the world. Knowledge of the DNA sequences of these viruses is important for oligonucleotide design to ensure traceability and proper calibration of assays, particularly where virus load determination is mandated.

### Nucleotide sequence accession numbers.

The sequence of the WHO B19V strain has been deposited in GenBank under the name WHO IRP, and the Ph. Eur. strain was deposited under the name EDQM BRP; the respective accession numbers are KM065414 and KM065415.
